# Validation of the hind feet position score and its association with heel height difference in dairy cows

**DOI:** 10.1007/s11259-024-10472-3

**Published:** 2024-07-27

**Authors:** Alexandra Hund, Anna Reiter, Johann Huber, Johann Kofler

**Affiliations:** 1 Department of Farm Animals and Veterinary Public Health, University Clinic for Ruminants, University of Veterinary Medicine Vienna, Vienna, Austria; 2 Agricultural Center for Cattle Production, Grassland Management, Dairy Food, Wildlife and Fisheries of Baden-Wuerttemberg (LAZBW), Aulendorf, Germany; 3 University of Veterinary Medicine Vienna, VetFarm Kremesberg, Pottenstein, Austria

**Keywords:** Leg score, Hind feet position score, Interdigital claw angle, Heel height, Cattle

## Abstract

Hind feet position scoring (HFPS) categorizes the outward rotation of the hind feet from the line parallel to the midline of the body in standing dairy cows. It has previously been used as an indication of both differences in heel height between the lateral and medial hind claw (to determine the need of hoof trimming) and the presence of claw lesions in sound cattle. In this observational study, the agreement of HFPS with other types of hoof angle measurements, as well as its association with the heel height difference (HHD) between the claws were investigated.

A total of 51 dairy cows were assessed independently by three observers in two measuring rounds on two different measuring days each. On each occasion, they scored the HFPS, and measured the angle of outward rotation of both hind feet using a digital protractor (DIG) and a compass app (COMP). Heel height difference was measured only during the second occasion. Intra- and interobserver agreement were calculated using weighted kappa statistics (HFPS) and intraclass correlation (DIG, COMP and HHD). Associations between HFPS and DIG, COMP and HHD were analyzed using linear mixed models.

Intra- and interobserver reliability were poor to good for HFPS, DIG, COMP and HHD. HFPS was significantly associated with DIG and COMP but not with HHD. Using the median value of repeated HFPS scores could increase the robustness of the HFPS assessment, as our data indicate that the cows frequently shift the position of their hind claws. Overall, there was a poor correlation between HHD and HFPS, so HFPS may not be determined by HHD alone; future research should consider other reasons for outward rotation of the hind feet.

**Supplementary Information:**

The online version contains supplementary material available at 10.1007/s11259-024-10472-3.

## Introduction

In cattle, there are anatomical differences between lateral and medial claws, with the lateral digit being longer in hindlimbs (Muggli et al. 2011). The lateral claw therefore carries more load, even in well-trimmed hind feet (Van der Tol et al. 2004); as chronic pressure on the dermis is believed to stimulate horn growth, this uneven weight distribution could result in an increased heel height of the lateral claw over time, further exaggerating the difference in weight load between the claws (Vokey et al. 2001; Oehme et al. 2019). Because of this growing difference in the heel height between the hind claws, the interdigital axis is assumed to rotate progressively outward over time (Holzhauer et al. 2005).

To evaluate differences in heel height and detect a need for its correction via hoof trimming, a visual hind feet position scoring (HFPS, or leg scoring) on the hind limbs of standing cows has been proposed (Bulgarelli-Jimenez et al. 1996). A HFPS score of 1 corresponds to the physiological angulation of the interdigital axis, parallel to the midline of the body. Under these conditions, Bulgarelli-Jimenez et al. (1996) assumed that the heel height of both claws of a hindlimb was approximately the same. In contrast, scores 2 and 3 are respectively considered as a moderately and severely deviant outward rotation of the hoof. During these conditions, the heel height of the lateral claw was assumed to be higher than the medial claw (Bulgarelli-Jimenez et al. 1996).

To our knowledge, the assumed correlation between an outwardly rotated hoof and differences in heel height between the claws have not been tested to date. Our primary goals were to validate the visual assessment of the HFPS against measurements of the interdigital angle measured with (1) a digital protractor (DIG) and (2) a compass app (COMP). Additionally, we wanted to evaluate the repeatability of the HFPS assessment and the DIG and COMP measurements. Our secondary goal was to verify whether the visual assessment of the HFPS is a suitable indicator of heel height difference (HHD) in the hind claws.

We hypothesized that the HFPS assessment would agree with the DIG and COMP measurements, and that there would be satisfactory intra- and interobserver reliability for all methods used to measure the degree of outward rotation of the hoof. Furthermore, we hypothesized that HFPS would be associated with HHD, and, since the cows were not trimmed between the two occasions when assessments were performed, that HFPS would increase in the animals between the two measuring days.

## Materials and methods

### Herd description

Of the 65 lactating cows at VetFarm Kremesberg, the Teaching and Research Farm of the University of Veterinary Medicine, Vienna, we selected 51 cows for our study. To focus on our main goal, the validation of HFPS by means of DIG and COMP, we decided to include only apparently healthy cows, i.e., cows that had not been identified as lame by the farm personnel. Therefore, we excluded lame cows, and those that had a block attached to one or more claws at the time of measurements and cows that had undergone hoof trimming during the five months prior to the start of the trial. The 51 remaining cows (*n* = 42 Fleckvieh, *n* = 9 Holstein Friesian) were kept together with the 14 excluded cows in a loose housing system with cubicles deep bedded with straw. A total of 72 lying and feeding places were available for the 65 cows. The walkways were rubber-matted and cleaned ten times per day using scrapers. The waiting area in front of the milking parlor and the outdoor paddock had a concrete floor. The cows received a total mixed ration, which was mixed and distributed eight times a day by a feeding robot.

In the year of the study, the mean annual milk yield of the selected cows was 9,133 kg, and the mean age of these cows was 5.3 (standard deviation (SD) ± 3.2) years. All cows in the herd were routinely subjected to functional hoof trimming twice a year. For animals in need of more frequent trimming (i.e., showing signs of lameness), one or two extra trims were performed per year.

### Data collection from cows

Three weeks before the start of the trial, the different scoring methods (HFPS) and measurement methods (DIG, COMP) were tested and trained at the VetFarm Kremesberg by the three observers. For HFPS assessment and the DIG and COMP measurement, the cows were restrained in the feeding fence next to each other to restrict lateral movement of the cows. During the entire measurement process, the cows were able to eat and were not manipulated by the three observers. Three additional people assisted the observers by recording the collected data and identifying the cows.

On the first day of the measurements on August 6 2020, all 51 cows were evaluated by all three observers in two separate runs. The observers were positioned behind the cows standing at the feeding fence. One observer collected the data of all cows using one method before switching to the next method to avoid self-validation. The order in which the cows were evaluated was changed between measuring methods and between both runs.

For the second round of measurements, the 51 cows were divided into four groups, each consisting of eleven to fourteen animals. The measurements took place on four different days (on October 13, October 20, November 3, and November 10, 2020). At these time points, in addition to the assessment of HFPS, DIG and COMP, the cows were also placed in lateral recumbency on a tilt table. This was done to measure the heel height difference (HHD) between the lateral and medial claw on both hindlegs, and to perform functional hoof trimming on all claws.

### Hind feet position scoring

**Fig. 1:**
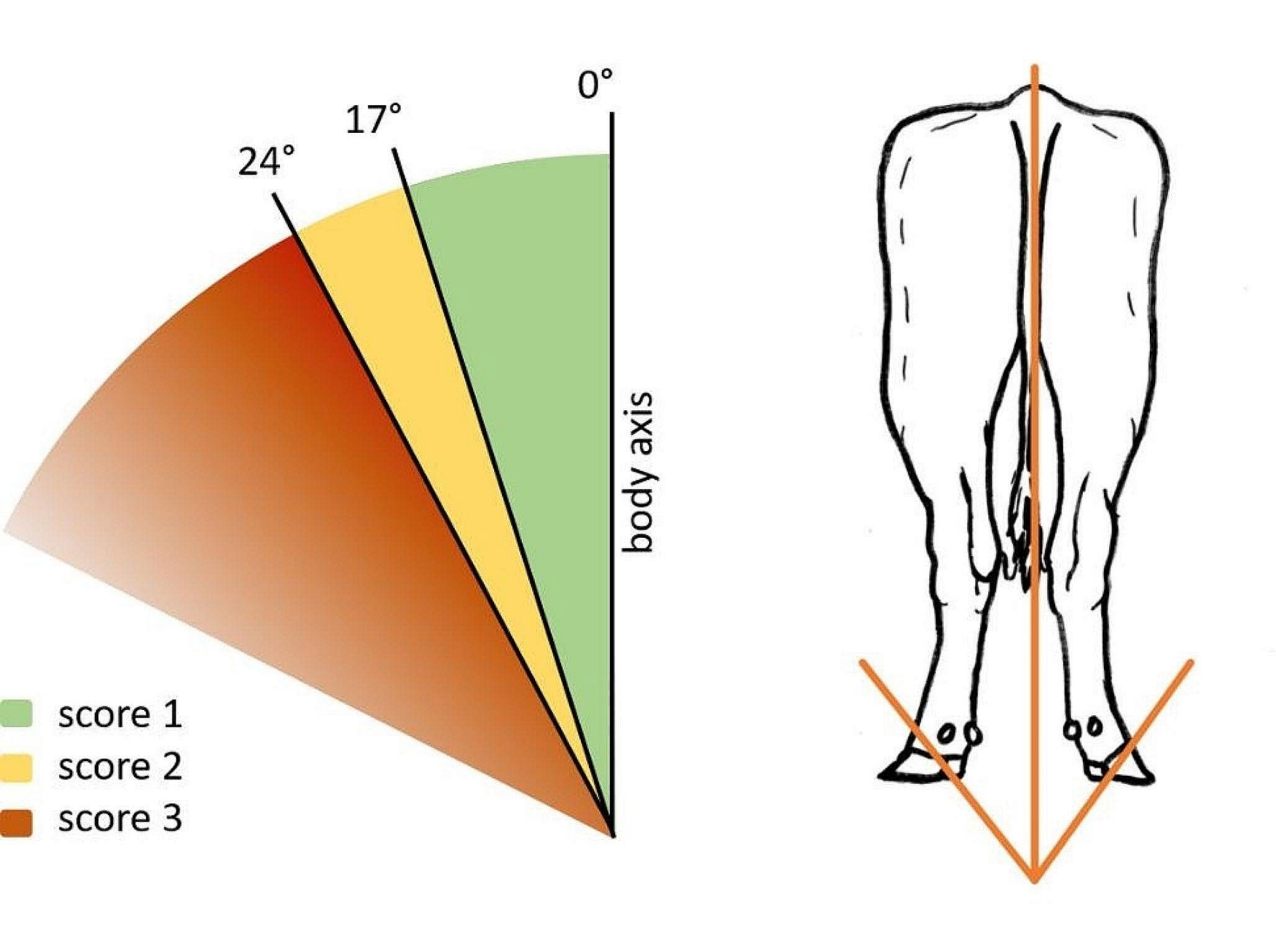
Schematic drawing for the assessment of the hind feet position score (HFPS): the body midline (0°) symbolizes the dorsal back line of the cow, and the second line passes through the interdigital space of the left and right hindlimbs

The assessment of HFPS involves evaluating the degree of external rotation of the left and right rear digits, determined by the angle between two imaginary lines, one running through the interdigital space of one pair of hind claws and the other running cranio-caudally along the length of the spinal column (= interdigital angle). A score of 1 was assigned for an angle < 17°, a score of 2 for an angle of 17° − 24°, and a score of 3 for an angle > 24° (Fig. 1 and Online Resource 1, Fig. 1) (Bulgarelli-Jimenez et al. 1996). We recorded a separate score for each hind limb.

### Measurement of the interdigital angle using a digital protractor

Similar to the HFPS assessment, the interdigital angle of the rear claws was measured using a digital (DIG) protractor (goniometer) with an LCD display (Walfront™ Electronics, Walmart, Sacramento, CA, USA). The device has a precision of ± 0.3°. The protractor device was held by the observers in front of their bodies and parallel to the ground. We did not define the height over the floor at which the device was held. One protractor leg was aligned with the extended dorsal midline of the cow, and the other protractor leg was aligned with the interdigital axis of one of the hind limbs. This angle measurement was performed successively on both rear claws.

The results of the DIG measurements contained one decimal and were rounded to whole numbers using the round function in R (rounding down to the lower number up to 0.5, and rounding up to the higher number from > 0.5).

### Measurement of the interdigital angle by means of a compass app

**Fig. 2:**
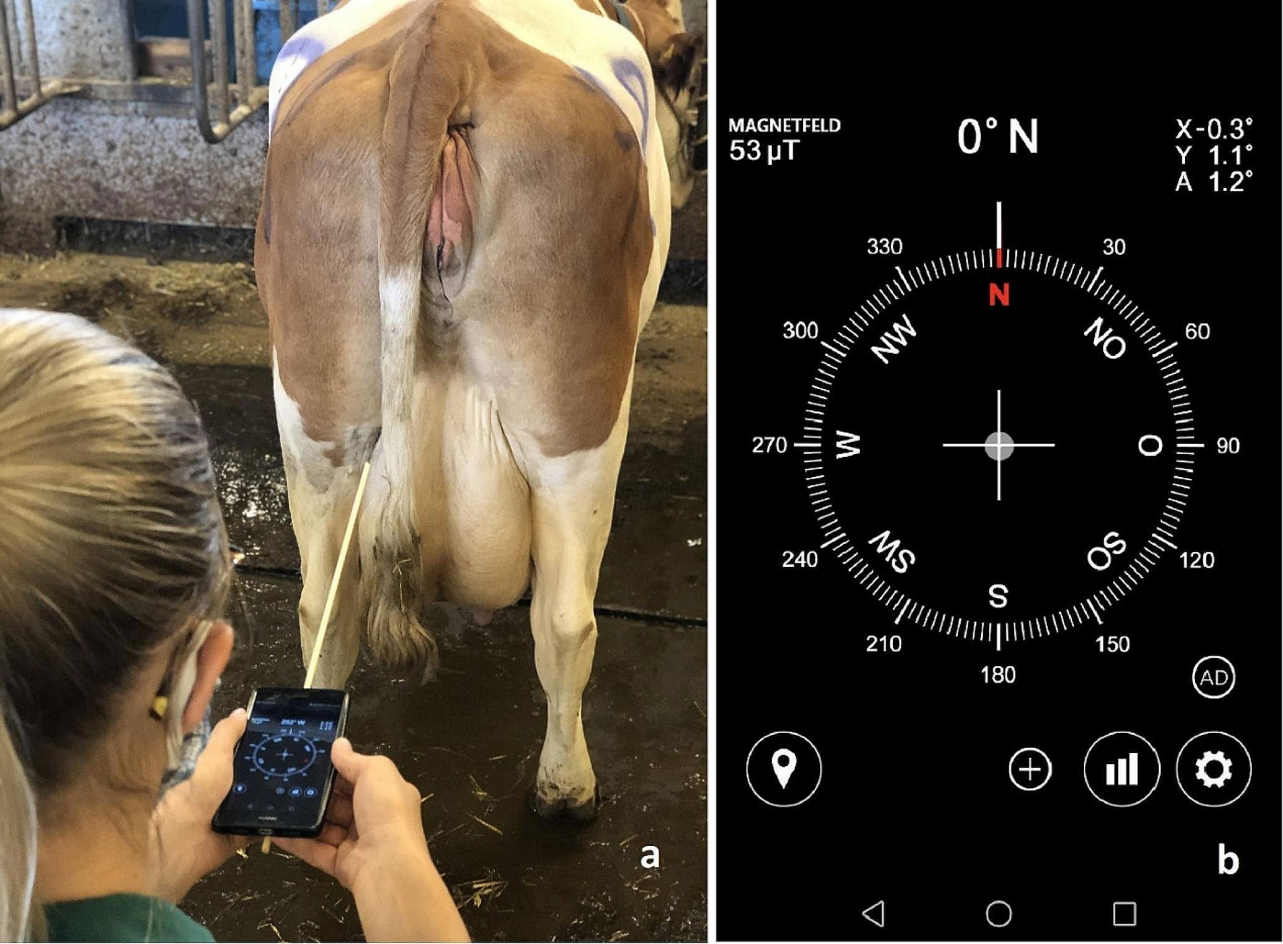
Measuring longitude and latitude using the compass app (COMP) on the smartphone. A wooden stick was attached to the back of the smartphone to support the measurement process (**a**). Screenshot of the compass app. In the center, there is a gray dot on the crosshairs, which ensures that the smartphone is held horizontally during the measurement process (**b**)

The observers used a compass app (COMP) for the Android systems (melon™ soft Compass, Seoul, South Korea) installed on their smartphones for the third measurement of the interdigital angle. Before each use, the compass was calibrated according to the instructions. To support the observers in aligning the measurement device with the body axes as described above, an approximately 50 cm long and 3 mm thick wooden stick was attached to the center of the back of the smartphone with adhesive tape and served as an extension of this centerline (Fig. 2). First, the reference direction (in degrees) of the cows’ dorsal midline was determined using the compass app, and then the degrees of the interdigital axis of the left and right rear claws were measured. The compass app provided a point with crosshairs in the center of the display to ensure the correct horizontal positioning of the device (Fig. 2). The two measured degrees per claw pair were subtracted, resulting in the compass angle measurement value COMP.

### Measurement of the heel height difference

**Fig. 3:**
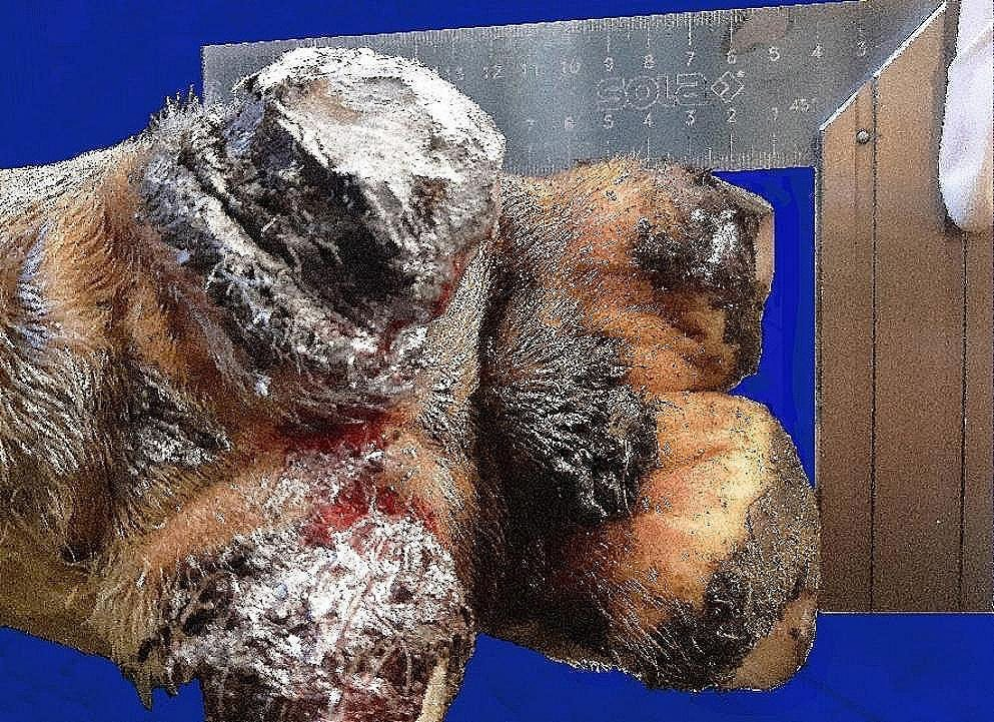
Measurement of the difference in the heel height (HHD) between the lateral and medial claws using a carpenter’s square; for this leg, the heel height of the lateral claw was 10 mm greater than that of the medial claw

At the time of the second measurements, HHD was measured at the rear claws using a carpenter’s angle (SOLA^®^ model SRB 250, SOLA-Messwerkzeuge GmbH, Götzis, Austria). The cows were placed in lateral recumbency on a tilt table, and the claws were cleaned. The HHD was determined only once by two observers. The reason for scoring this outcome less times was that it we wanted to avoid restraining the animals more than once, which made it impossible to obtain two unrelated measurements from the same observer. The number of observers was reduced to keep the time on the tilt table as short as possible for the cows. For the HHD measurement, the short leg of the carpenter’s square was placed on the caudal aspect of the sole of the claw with the greater heel height in such a way that the long leg of the carpenter’s square rested laterally and abaxially against the claw with the lower heel height while maintaining right angles to the interdigital axis. The HHD was then measured in mm from the long leg of the carpenter’s angle (Fig. 3).

### Statistical analyses

The necessary sample size to show that both DIG and COMP agreed with HFPS was calculated using G*Power, version 3.1 (Faul et al. 2009). We assumed that the interdigital angles ranged from 0 to 45 degrees, that the difference between measurement methods would be ± 10° and that we would consider a maximum difference of 5° between methods to agree with the measurement results. Thus, with a power of 80%, an alpha of 5% and an omega of 0.5 and a tolerance of 5° for the interdigital angle measurements, at least 32 cows had to be included in the study.

For the statistical evaluation, data from 51 cows were used. Data analysis was conducted using R (R Foundation for Statistical Computing 2020, Vienna, Austria) and RStudio (R Studio Team 2020, Vienna, Austria) with the ggplot2 and dplyr packages (Wickham 2016; R Foundation for Statistical Computing 2020; R Studio Team 2020; Wickham et al. 2020).

Intra-observer percent agreement was calculated for both DIG and COMP with a tolerance of 3 degrees, because we assumed that humans could differentiate angles with this interval. Intra- and interobserver agreement was calculated for pair-wise comparisons of the ordinal HFPS data per foot and observer comparing run 1 to run 2 using square-weighted kappa in the package psych (Revelle 2024). Kappa values of ≤ 0.40 were considered poor, values of 0.41–0.60 were considered moderate, values of 0.61–0.80 were considered substantial, and values of 0.81–1.0 were considered almost perfect agreement (Landis and Koch 1977).

Intraclass correlation (ICC) (model = “twoway,” type = “consistency,” unit = “single”) was calculated for the continuous data of angles measured by DIG, COMP, and for the HHD using the package ICC (Wolak et al. 2012). ICC values less than 0.5, between 0.5 and 0.75, between 0.75 and 0.9, and greater than 0.90 were considered indicative of poor, moderate, good, and excellent reliability, respectively (Koo and Li 2016).

To calculate percent agreement in HHD between the two observers we decided to accept a tolerance of 3 mm because we assumed that it was reasonable to be accurate within those limits under the circumstances the measurement was performed.

When analysing the association between HFPS, DIG and COMP, and their association with HHD, we used aggregated data. For HFPS, DIG and COMP, there were 12 values per animal per method on each measuring day (3 observers * 2 runs * 2 claw pairs), which we aggregated into one value per cow and day. In HFPS we aggregated the data of one day per cow to a median value, and a mean value in DIG, COMP and HHD. The associations between HHD, DIG, and COMP, and their associations with HFPS were analyzed using linear mixed models using function lmer in package lme4 (Bates et al. 2015). We applied a linear mixed-effects model with maximum likelihood estimation for the aggregated COMP values per animal and measuring day as outcome variable. As explanatory variable we fitted the mean DIG value per animal and measuring day (model A). Similarly, in models B and C we tested the effects of the aggregated HFPS data (median per measuring day and animal) as explanatory variable on the outcome variables DIG (B) and COMP (C). In models D, E and F we fitted the aggregated values of DIG (D), COMP (E) or HFPS (F) as explanatory variable to test their effect on HHD as outcome variable. In all models animal ID was included as grouping factor to account for the two measuring days.

The models took the general format

$$Yi\;\sim\;N(\eta i,\sigma2)$$

$$Yimj\:=\:\beta0\:+\:\beta1,m(i)\:+\:bj(i)$$

$$bj\;\sim\;N(0,\sigma2b)$$

where *Y*_*i*_ was the outcome variable, m(i) the explanatory variable, j(i) the animal ID corresponding to the ith observation, and β0 the intercept value.

For further information, please see Online Resource 2. An association was considered significant if *p* < 0.05.

## Results

### Hind feet position: intra- and interobserver agreement of three assessment methods

For all assessments of HFPS performed on both measuring days (*n* = 1,224), the observers predominantly recorded score 1 (*n* = 728; 59.5%) and score 2 (*n* = 400; 32.*7*%), whereas score 3 was documented only 96 times (7.8%). Regarding the difference between runs within the same day, the intraobserver percent agreement was 69.3% of HFPS (run 1–run 2 = 0, *n* = 424), 29.7% (run 1–run 2 = |1|, *n* = 182) differed by 1 score and 1.0% differed by 2 scores (run 1–run 2 = |2|, *n* = 6). The intraobserver weighted kappa values ranged from 0.45 to 0.66 for all observers on measuring day 1 and from 0.29 to 0.75 on day 2. The interobserver weighted kappa values were somewhat lower and ranged from 0.37 to 0.54 on day 1 and from 0.34 to 0.60 on day 2 (see Table 1 for more details). Using the median value of both runs and both legs per observer, cow and measuring day, the agreement between the observers improved to kappa values between 0.51 (95% CI 0.36–0.65) and 0.67 (95% CI 0.51–0.83).

**Table 1:** Intra- and interobserver agreement of Hind feet position score (HFPS) of all three observers on two measuring days in two runs each (kappa weighted squared)

	Measuring day 1			Measuring day 2		
**HFPS**	*Observer 1*	*Observer 2*	*Observer 3*	*Observer 1*	*Observer 2*	*Observer 3*
*Right leg*						
Observer 1	*0.45* *[0.21–0.68]*	0.52 [0.32–0.72]	0.47 [0.29–0.65]	*0.59* *[0.35–0.83]*	0.43 [0.24–0.62]	0.49 [0.25–0.72]
Observer 2	**0.51** **[0.30–0.71]**	*0.52* *[0.29–0.74]*	0.46 [0.20–0.71]	**0.44** **[0.20–0.68]**	*0.75* *[0.58–0.91]*	0.29 [0.07–0.50]
Observer 3	**0.40** **[0.15–0.64]**	**0.37** **[0.11–0.62]**	*0.66* *[0.51–0.80]*	**0.34** **[0.12–0.56]**	**0.43** **[0.20–0.66]**	*0.29* *[0.01–0.57]*
*Left leg*						
Observer 1	*0.49* *[0.29–0.68]*	0.42 [0.22–0.61]	0.45 [0.23–0.66]	*0.54* *[0.32–0.76]*	0.40 [0.17–0.63]	0.60 [0.41–0.79]
Observer 2	**0.42** **[0.16–0.68]**	*0.47* *[0.26–0.69]*	0.54 [0.30–0.78]	**0.54** **[0.36–0.71]**	*0.74* *[0.62–0.86]*	0.43 [0.20–0.65]
Observer 3	**0.45** **[0.23–0.67]**	**0.39** **[0.19–0.59]**	0.60 [0.44–0.77]	**0.41** **[0.17–0.66]**	**0.36** **[0.10–0.61]**	0.41 [0.14–0.69]

Annotation: square brackets = 95% confidence interval; values in italics = intraobserver reliability; values in bold = run 2. Kappa values ≤ 0.40 poor agreement, 0.41–0.60 moderate agreement, 0.61–0.80 substantial agreement, > 0.81 almost perfect agreement

Of the 1224 DIG measurements taken, 68.3% (*n* = 836) were below 17 degrees, 28.6% (*n* = 350) between 17 and 24 degrees, and 3.1% (*n* = 38) over 24 degrees.

**Fig. 4:**
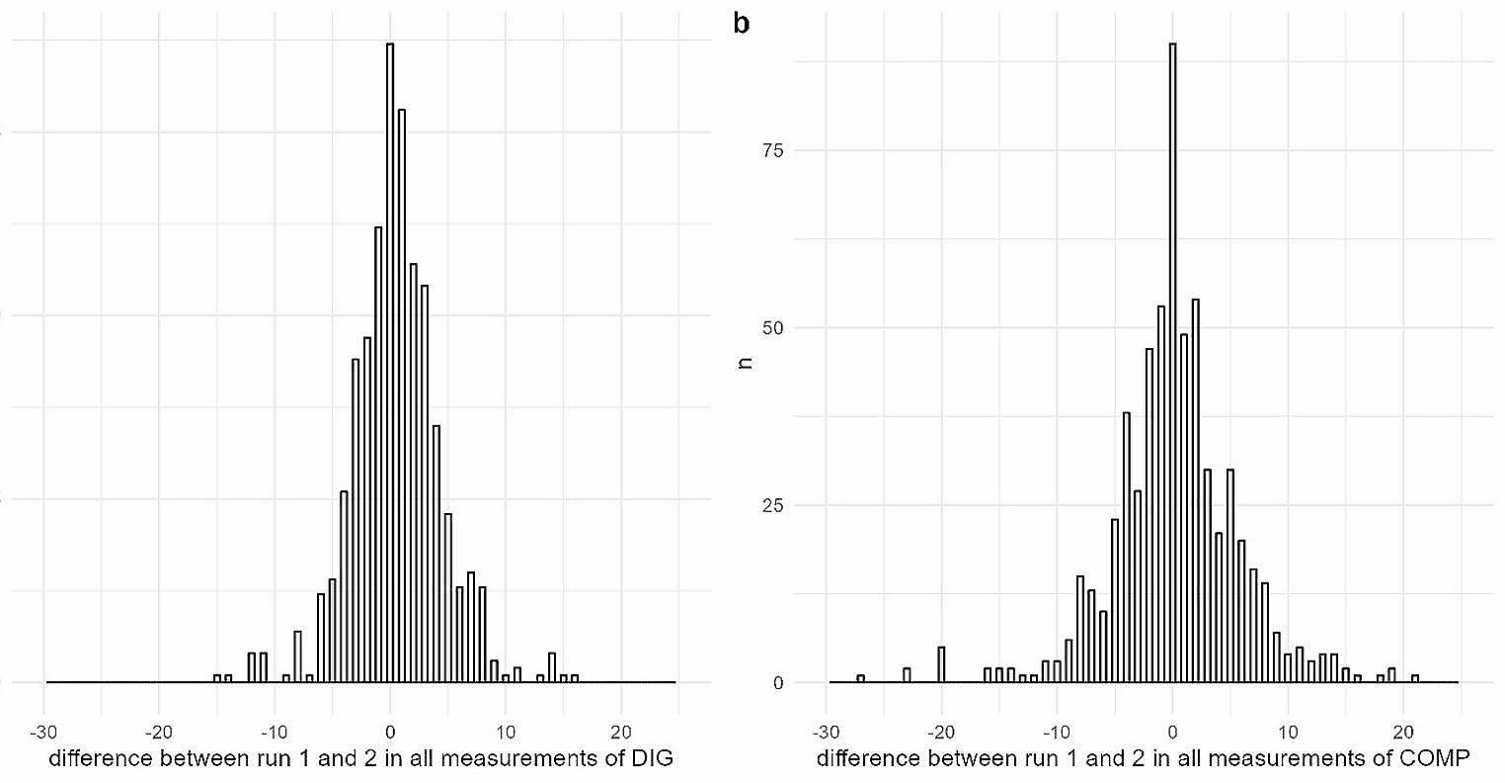
Difference between the interdigital angle measurements taken with a digital protractor (**a**, DIG) and using a compass app (**b**, COMP) in two runs on 51 cows by three observers. The difference shown is the angle measured by one observer in run 1 subtracted by the angle measured in run 2 on the same day by the same observer. Measurements were performed on two different days 10–14 weeks apart; data from both days is shown in the figure

The difference in the measured outward rotation per leg and observer ranged from − 15 to 16 degrees between DIG runs performed on the same day (Fig. 4a). Given our tolerance of 3 degrees, 70.1% (*n* = 429 of 612 values) of the DIG measurements were in agreement for the two runs within the same day and observer.

The intra- and interobserver intraclass correlation coefficient for DIG are shown in Table 2.

**Table 2:** Intraclass coefficient as a measure for intra- and interobserver reliability of measurements of the interdigital angle using a digital protractor (DIG) on two measuring days in two runs each by three observers

Measuring day 1				Measuring day 2		
**DIG**	Observer 1	Observer 2	Observer 3	Observer 1	Observer 2	Observer 3
*Right leg*						
Observer 1	*0.63* *[0.43–0.77]*	0.54 [0.31–0.71]	0.40 [0.19–0.61]	*0.62* *[0.42–0.77]*	0.56 [0.33–0.72]	0.38 [0.12–0.60]
Observer 2	**0.38** **[0.12–0.59]**	*0.83* *[0.73–0.90]*	0.32 [0.05–0.54]	**0.38** **[0.12–0.6]**	*0.82* *[0.71–0.90]*	0.29 [0.02–0.52]
Observer 3	**0.47** **[0.23–0.66]**	**0.27** **[-0.01–0.50]**	*0.69* *[0.51–0.81]*	**0.48** **[0.23–0.66]**	**0.25** **[-0.02–0.49]**	*0.69* *[0.51–0.81]*
*Left leg*						
Observer 1	*0.47* *[0*,*22–0*,*66]*	0.25 [-0,02–0,49]	0.37 [0.1–0.58]	*0.45* *[0.20–0.64]*	0.27 [-0.01–0.51]	0.36 [0.10–0.58]
Observer 2	**0.41** **[0.15–0.62]**	*0.67* *[0*,*49–0*,*80]*	0.21 [-0.07–0.45]	**0.40** **[0.14–0.61]**	*0.68* *[0.49–0.80]*	0.20 [-0.01–0.45]
Observer 3	**0.23** **[-0.05–0.47]**	**0.39** **[0.14–0.60]**	0.73 [0.56–0.83]	**0.24** **[-0.04–0.48]**	**0.38** **[0.12–0.59]**	0.73 [0.57–0.84]

Annotation: square brackets = 95% confidence interval; values in italics = Intraobserver reliability; values in bold = run 2. ICC values < 0.5 poor agreement, 0.5 - 0.75 moderate agreement, 0.75 - 0.9 good agreement, > 0.90 excellent agreement

Of the total 1224 COMP measurements from both days, 76.3% (*n* = 934), 20.4% (*n* = 250) and 4.9% (*n* = 60) were below 17 degrees, between 17 and 24 degrees and above 24 degrees, respectively.

The difference in measured outward rotation per leg and observer ranged from − 27 to 21 degrees between the COMP runs performed on the same day (Fig. 4b). Given our tolerance of 3 degrees, 57.2% (*n* = 350 of 612 values) of the COMP measurements were in agreement for the two runs within the same day and observer.

The ICC for the intra- and interobserver agreement of the measured COMP values between the first and second runs within the same measuring day are shown in Table 3.

**Table 3:** Calculation of intra- and interobserver agreement using the ICC for the interdigital angle values revealed by the compass app (COMP) by the three observers on two measuring days in two runs each

Measuring day 1				Measuring day 2		
**COMP**	*Observer 1*	*Observer 2*	*Observer 3*	*Observer 1*	*Observer 2*	*Observer 3*
*Right leg*						
Observer 1	*0.39* *[0.13–0.60]*	0.53 [0.30–0.70]	0.55 [0.33–0.72]	*0.58* *[0.37–0.74]*	0.44 [0.19–0.64]	0.43 [0.18–0.63]
Observer 2	**0.16** **[-0.12–0.42]**	*0.46* *[0.21–0.65]*	0.40 [0.14–0.61]	**0.29** **[0.01–0.52]**	*0.55* *[0.33–0.72]*	0.42 [0.16–0.62]
Observer 3	**0.13** **[-0.15–0.39]**	**0.19** **[-0.09 -0.44]**	*0.56* *[0.34–0.72]*	**0.47** **[0.22–0.66]**	**0.47** **[0.23–0.66]**	*0.78* *[0.64–0.87]*
*Left leg*						
Observer 1	*0.47* *[0.22–0.66]*	0.57 [0.36–0.73]	0.41 [0.16–0.62]	*0.52* *[0.29–0.69]*	0.48 [0.24–0.67]	0.29 [0.02–0.52]
Observer 2	**0.43** **[0.18–0.63]**	*0.54* *[0.31–0.71]*	0.58 [0.37–0.74]	**0.42** **[0.17–0.63]**	*0.48* *[0.24–0.67]*	0.45 [0.2–0.64]
Observer 3	**0.35** **[0.09–0.57]**	**0.51** **[0.28–0.69]**	*0.69* *[0.52–0.81]*	**0.37** **[0.11–0.59]**	**0.57** **[0.36–0.73]**	*0.56* *[0.34–0.73]*

Annotation: square brackets: 95% confidence interval values in italics = intraobserver match; values in bold = run 2. ICC values < 0.5 poor agreement, 0.5–0.75 moderate agreement, 0.75–0.9 good agreement, > 0.90 excellent agreement

**Fig. 5:**
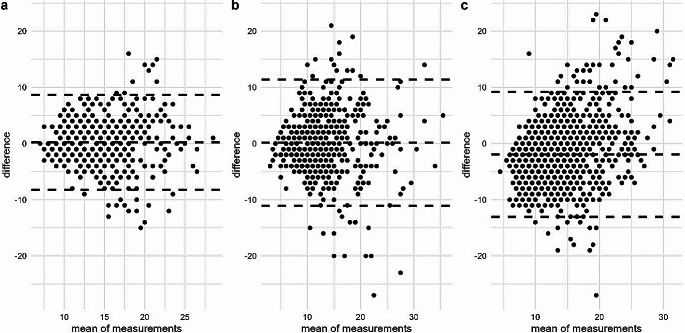
Bland‒Altmann plot of the difference between runs of measurements of DIG (**a**) and COMP (**b**) and between the measurement methods DIG and COMP (**c**): Each pair of DIG and COMP values were measured on one leg by one observer during two runs of measurements on the same day, while the comparison between the DIG and COMP values was performed within the same leg, run and observer. The difference between values is evaluated by plotting the difference between the values on the y axis relative to the mean of both values on the x-axis

Bland‒Altmann plots were used to explore the differences within observer and measuring day between runs of the same measuring method, and between the DIG and COMP values in the same runs (Fig. 5).

### Relation of hind feet position score and angles measured using a digital protractor and a compass app

**Fig. 6:**
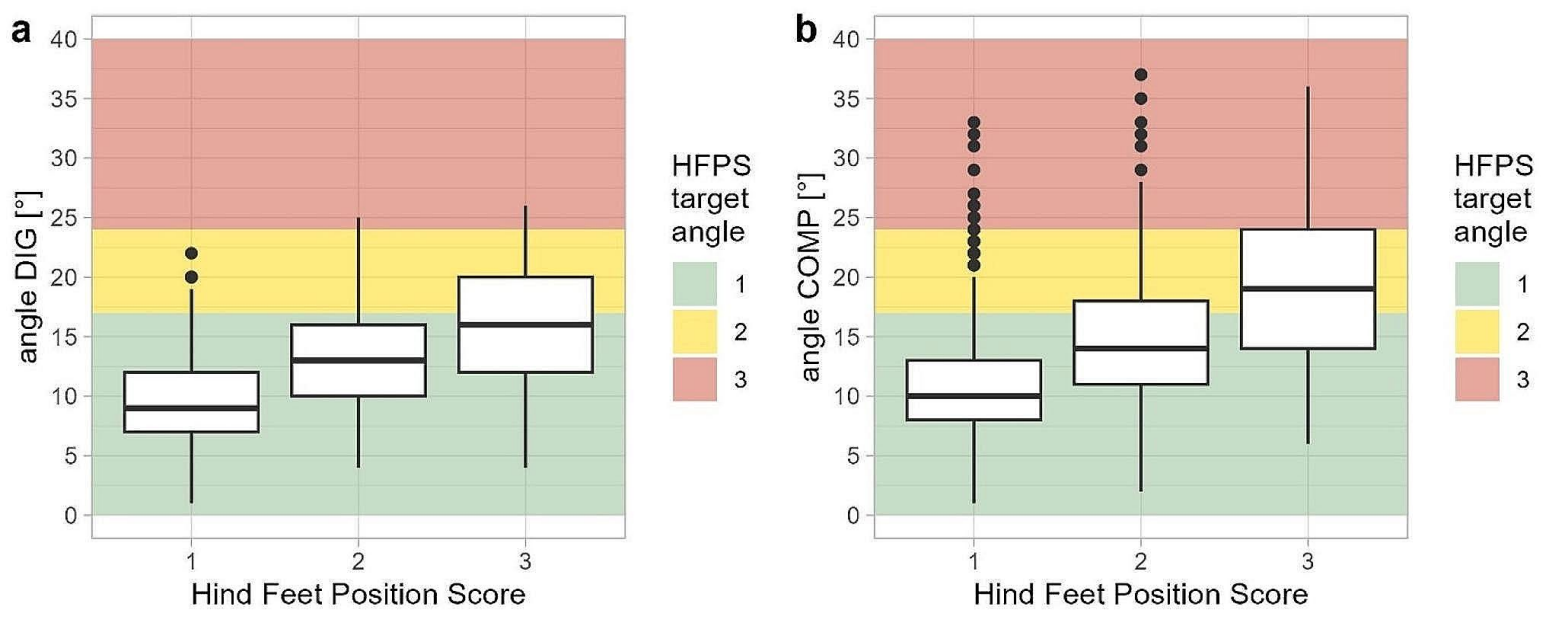
Boxplots showing the distribution of the outward rotation of the hoof, measured with a digital protractor (DIG, **a**) and a compass app (COMP, **b**), in relation to the hind feet position score (HFPS) assessed in the same run by the same rater on the same hindlimb. Each cow was measured in two runs on each of the two measuring days, 10–14 weeks apart. The figure shows data from all runs. The background colours show the cut-off values of the three HFPS, as defined by Bulgarelli-Jiménez (1996)

The relationship between DIG and COMP, was highly significant (model A in Online Resource 2, *p* < 0.001), suggesting a close similarity between the DIG and COMP values. Similar associations were found between both HFPS and DIG (model B in Online Resource 2, *p* < 0.001), and between HFPS and COMP (model C in Online Resource 2, *p* < 0.001). However, the degrees of the interdigital angle measured by DIG and COMP did not align with the definitions of the HFPS scores, as the observers tended to overestimate the outward rotation of the claw when using the HFPS scale. In other words, the raters often gave a HFPS of 3 to animals when their DIG and COMP values instead suggested a HFPS of 1 or 2. For example, the median of DIG values measured in cows with a HPFS of 3 was below 17 degrees, corresponding to a HFPS of 1 (Fig. 6).

### Heel height difference

Differences in heel height between the lateral and medial claws ranged from 0 to 15 mm. The mean HHD was 8.4 mm (± 2.9 mm SD) in the left rear claws and 5.9 mm (± 2.66 mm SD) in the right rear claws. For the left hindlimb, all measurements (*n* = 102; 51 cows measured by two observers) showed that the heel on the lateral claw was higher. For the right hindlimb, 94 measurements showed a higher heel height on the lateral claw, while 4 showed the same heel height for both claws and 4 showed a higher heel height for the medial claw. Both observers agreed that the heel of the medial claw was higher in two cows, and agreed that the heel height was the same in the lateral and medial claw in one cow. In two cows, one observer measured no difference in heel height, while the other observer measured a difference of 1 mm.

Differences in the measured heel height between the two observers ranged from − 6 to 6 mm. With a tolerance of 3 mm, 80.39% of the values (*n* = 82 of 102) were consistent between the observers. The ICC was 0.76 (95% CI 0.62–0.86) for the left digit and 0.72 (95% CI 0.55–0.83) for the right digit, indicating moderate to good agreement (Koo and Li 2016).

### Hind feet position score and heel height difference

**Fig. 7:**
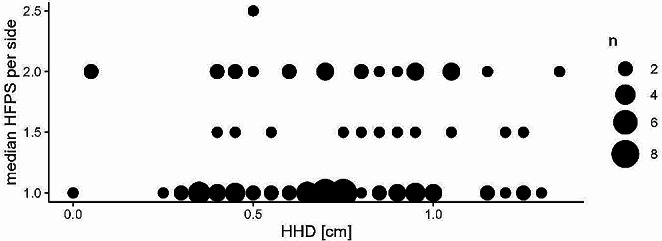
Scaled scatterplot showing the relationship between the mean heel height difference (HHD) and the median hind feet position score (HFPS) using all measurements performed on both hind legs by both observers. The size of the points represents the number (n) of times a specific value occurred in the data set

Hind feet position score was not significantly related to HHD (model F (Online Resource 2), *p* = 0.846). Figure 7 shows that most data points of increasing HHD were measured in cows with HFPS of 1. HHD was also not related to the interdigital angles measured with DIG (model D (Online Resource 2), *p* = 0.836) or COMP (model E (Online Resource 2), *p* = 0.236).

### Hind feet position score: changes between measuring days

**Fig. 8:**
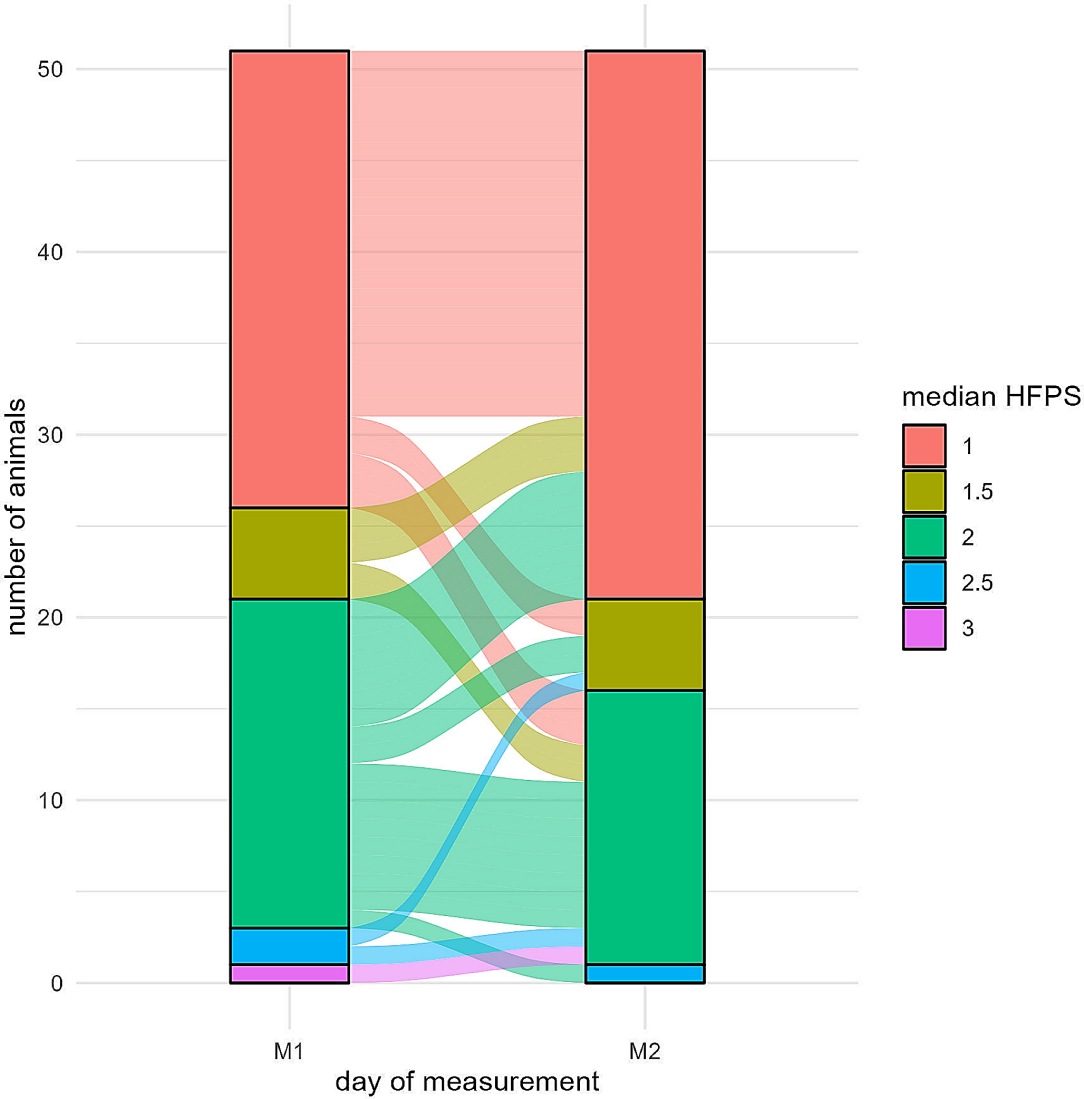
Sankey plot showing the change in the hind feet position score (HFPS) between two measuring days 10–14 weeks apart (M1 = measuring day 1, M2 = measuring day 2). The value for an individual cow on each day are the median value of all measurements obtained from both hind legs by all three observers during the two measuring rounds on the same day (in total 12 values)

The two measuring days when HFPS was assessed were 10 to fourteen weeks apart. Figure 8 shows the change in HFPS for each animal between the measuring days. A paired Wilcoxon test showed no significant difference between the median HFPS at the two time points (*p* = 0.085, z-value = -1.724).

## Discussion

To date, only a few researchers have used hind feet position scoring to detect animals with differences in heel height, or as a proxy for lameness (Bulgarelli-Jimenez et al. 1996; Zurbrigg et al. 2005). Recently, researchers have shown that a model containing HFPS in combination with other data (animal data, milk performance records, farm risk factors, AMS data, sensor data) could predict lameness in dairy cattle with the highest accuracy (sensitivity 0.725, specificity 0.775); Locomotion score was significantly correlated with HFPS (rs: 0.394, *p* < 0.0001) (Lemmens et al. 2023). Furthermore, it was used as an auxiliary trait for the evaluation of heritability of lameness in dairy cows (Koeck et al. 2024). The latter study showed that sensitivity and specificity of HFPS for detecting lameness defined by locomotion score ≥ 2 were 69.5% and 66.8%, respectively, and that the genetic correlation between those traits was 0.80. However, the phenotypic correlation was only 0.38, suggesting that lameness cannot be reliably detected using HFPS.

Intraobserver reliability of HFPS in the current study ranged from 0.29 to 0.75, and interobserver reliability from 0.34 to 0.6, indicating substantial intra- and moderate interobserver agreement at most. Holzhauer et al. (2005) reported slightly lower reliability of HFPS, called ‘leg score’ by the authors: In their study the interobserver reliability was somewhere between poor and moderate, while the intraobserver reliability was somewhere between poor and substantial (Landis and Koch 1977).

According to our data, combining multiple observations of HFPS and using the median of all observations of both hind legs by an observer on a given measuring day improved the interobserver agreement, indicating that multiple observations of HFPS may yield more reliable results. Capion at al. (2008) also found that scores for outward rotation of the claw or leg distal to the hocks were rather dynamic and suggested that several scorings of individual cattle appear to be needed to classify cattle correctly for this trait.

Even though the percent agreement between runs of DIG and COMP was around 70% and 57.2% (given a tolerance of 3%), the interdigital angle measurements showed considerable variation between runs in some animals (up to 27 degrees) – even when measured by the same observer. Additionally, moderate and poor intra- and interrater reliability was found also for DIG and COMP.

The lack of good agreement between runs could result from the cows shifting their weight frequently and thus changing the degree of outward rotation between measurements, or from difficulties in assessing the correct outward rotation by the observers. We identified several practical challenges with our measuring methods, where particularly DIG became more demanding as the interdigital angle increased. The observer had to step closer to the cow for the digital protractor to form an extension of the line through the interdigital space, which made it more difficult to correctly determine both reference lines. The indirect determination of the interdigital angle by means of COMP was comparatively easier since the reference lines were much easier to determine. However, with a compass, which is usually used to display the direction of the Earth’s magnetic field (Renner 2022), measurement disturbances due to environmental influences or other magnetic fields, such as a magnet inserted into the cow’s reticulum for foreign body prevention/treatment, cannot be completely excluded.

Additionally, for all types of measurements of interdigital angles (HFPS, DIG and COMP), a certain amount of visual judgment was necessary to establish the two imaginary lines of the angle, which could thus affect the objectivity of the angular measurements. Moreover, the measurements could not be performed on an animal by all three observers at the same time, adding another layer of complexity to the observer agreement. Due to observer drift, an observer may assess the first cow of the first run using the first method differently than the last cow using the last method, but the first and last cow and method were not the same for all observers (Smith 1989).

One other aspect of the evaluation of HFPS is the ability of humans to correctly judge angles in natural scenes (Kim and Burge 2018). Angle perception is an important visual process, and several theories have been developed to explain its mechanism in the human mind (Xu et al. 2018). In short, angle perception depends on a number of factors, such as the global shape of the stimuli or the size of the angle. Interestingly, Weber’s law, meaning that the precision of our perceptual judgment decreases when the magnitude of the sensory attribute in question increases, does not seem to apply to angle vision, as it fluctuates at several points (Xu et al. 2018). Hence, we cannot make any judgment about the accuracy of the ability of our observers to correctly assess, differentiate and categorize interdigital angles.

Weight shifting, another possible explanation for the high proportion of only poor to moderate intra- and interobserver reliabilities, has been used as a behavioral indicator to detect lameness in cows kept in tie-stalls (Leach et al. 2009). Another cause of weight shifting could be the time of restraint in the headlocks, which we did not record in our study. Cooper et al. (2008) showed that weight shifting was a response to the deprivation of lying and deemed it indicative of discomfort and frustration; it enabled cows to cope with forced standing by alleviating strain on the legs and hoofs.

Given the limitations discussed above, single values of neither DIG nor COMP can serve as gold standards for measuring the outward rotation of the claw. A more sophisticated method to repeatedly determine interdigital angles over a period of time is needed to make more accurate measurements of the “true” angle, which may not even exist. We found that the differences between DIG and COMP measurements seemed to increase with increasing interdigital angles, as shown in Fig. 5. This finding suggests that particularly cows with large outward rotation of the claws may have shifted their weight and altered the rotation of their claw axis considerably between measurements.

There was a significant relationship in our study between the HFPS and the DIG and COMP measurements. However, when comparing the DIG and COMP values against the HFPS obtained in the same run by the same observer, the number of cows with HFPS 2 and 3 were overestimated. We suggest that this systematic bias may be due to the disproportionately high proportion of cows with HFPS 1 on this farm, which could have led to the observers becoming accustomed to seeing normal interdigital angles and therefore overestimating the deviations they observed. Furthermore, all three observers value foot health in cattle and might therefore reacted more strongly to animals with outward rotation of the claw. Given that the cows in our study had not been trimmed for at least seven months when HHD was measured, the substantial difference we observed in heel height was expected. The magnitude of HHD in our study aligns with that reported by others (Nuss 2006; Mohamadnia 2013).

More surprisingly, our data does not support that HFPS is associated with HHD. However, it should be noted that due to our exclusion criteria, the interpretation of our results are limited to cows with relatively normal feet position, as most of the cows scored 1 for HFPS. The lack of association between HFPS and HHD could indicate that outward rotation of hind claws may be caused by other factors than HHD, such as poor conformation in the tarsal joint (Leach et al. 2009). Leach et al. (2009) reported that a deviant claw angle when standing was not associated with lameness in tie-stall housed cows, and that they observed outward rotation of the feet in sound cows as often as they did in lame cows. However, the authors suggested that an outward rotation of the claw could be associated with so early stages of lesion development that no effects on locomotion could be observed and recommended further investigation into this aspect. This suggestion is indirectly supported by the findings of Zurbrigg et al. (2005), who reported that tie-stall housed cows that had less than 20⁰ outward rotation of their hind claws when standing were more likely to stand with a straight back line (arched back line in standing cows is a lameness indicator; Sprecher et al. 1997), compared to cows with a more deviant hoof position. So rather than being due to HHD, the rotation might be an active movement of the cow to take weight off a painful lateral claw. Further research should therefore evaluate a potential association between outwardly rotated hooves and presence of claw lesions.

Other reasons for the rotation might be poor conformation (mild to moderate valgus malalignments in the tarsal joints) or a large udder, which has been shown to influence locomotion scores (Leach et al. 2009; Chapinal et al. 2009).

In our study, HFPS did not increase over ten to 14 weeks without foot trimming. This is in contrast with Telezhenko et al. (2009), who showed that the toe length, which is positively correlated with the heel height (Vokey et al. 2001), of the lateral claw grew to exceed that of the medial claw for animals housed on slatted concrete and solid rubber floors during their study period of an average of 174 d. The authors attributed the increasing difference in toe length to a higher wear rate in the medial compared to the lateral rear claw. However, Vokey et al. (2001) found no difference in wear between medial and lateral claws but showed that the growth of the lateral claw was more rapid in cows kept on concrete alley surfaces or concrete stalls compared to rubber alleys and sand mattresses. Moreover, Vokey et al. (2001) also reported that higher lateral heel height was associated with more severe lesions in all hind claws.

In conclusion, we found moderate intrarater agreement of HFPS, DIG and COMP, and poor (DIG and COMP) to moderate (HFPS) interrater reliability. These findings may have been influenced by visual biases when assessing HFPS, technical difficulties in handling the measurement devices and frequent weight shifting in the cows. However, averaging multiple repeated measurements resulted in better interobserver reliability. There are some indications that cows with highly variable measurements could be promising candidates for hoof trimming, as variable measurements suggest frequent weight shifting which could indicate discomfort from the claws. DIG and COMP were significantly associated with HFPS, but the degree of outward rotation was generally overestimated by the observers when using HFPS. This suggests that HFPS was the most biased assessment method, and that DIG or COMP might be more objective methods to determine the degree of outward rotation. We found no significant associations between HFPS and HHD, suggesting that HHD may not be the only reason for outward rotation of the hind feet.

## Supplementary Information

Supplementary Material 1.

Supplementary Material 2.

## Data Availability

No datasets were generated or analysed during the current study.
