# Correction: Validation of the hind feet position score and its association with heel height difference in dairy cows

**DOI:** 10.1007/s11259-024-10495-w

**Published:** 2024-08-09

**Authors:** Alexandra Hund, Anna Reiter, Johann Huber, Johann Kofler

**Affiliations:** 1 Department of Farm Animals and Veterinary Public Health, University Clinic for Ruminants, University of Veterinary Medicine Vienna, Vienna, Austria; 2 Agricultural Center for Cattle Production, Grassland Management, Dairy Food, Wildlife and Fisheries of Baden-Wuerttemberg (LAZBW), Aulendorf, Germany; 3 VetFarm Kremesberg, University of Veterinary Medicine Vienna, Pottenstein, Austria

**Correction: Veterinary Research Communications**

10.1007/s11259-024-10472-3

The wrong Supplementary file was originally published with this article; it has now been replaced with the correct file.

