# Supplementary material for: Validation of the hind feet position score and its association with heel height difference in dairy cows
Source: Vet Res Commun. 2024 Jul 27;48(5):3073–85. doi: 10.1007/s11259-024-10472-3 (PMC11442649; doi:10.1007/s11259-024-10472-3)
Supplement: Supplementary file 2 — Supplementary Material 2. [file 11259_2024_10472_MOESM2_ESM.pdf]

Model Nr.	Model type	Data aggregation	Formula
**A**	Linear mixed model (lmer)	DIG: Mean of all scores per cow and day<br>COMP: Mean of all scores per cow and day	$y_{imj} = \beta_0 + \beta_{1,m(i)} + b_{j(i)}$<br>Y: COMP, m(i): DIG, j(i): animal ID corresponding to the ith observation
**B**	Linear mixed model (lmer)	DIG: Mean of all scores per cow and day<br>HFPS: Median of all scores per cow and day	$y_{imj} = \beta_0 + \beta_{1,m(i)} + b_{j(i)}$<br>Y: DIG, m(i): HFPS, j(i): animal ID corresponding to the ith observation
**C**	Linear mixed model (lmer)	COMP: Mean of all scores per cow and day<br>HFPS: Median of all scores per cow and day	$y_{imj} = \beta_0 + \beta_{1,m(i)} + b_{j(i)}$<br>Y: COMP, m(i): HFPS, j(i): animal ID corresponding to the ith observation
**D**	Linear mixed model (lmer)	HHD: Mean of all measurements of one leg<br>DIG: Mean of all measurements of DIG of one leg on the same day (measuring day 2)	$y_{imj} = \beta_0 + \beta_{1,m(i)} + b_{j(i)}$<br>Y: HHD, m(i): DIG, j(i): animal ID corresponding to the ith observation
**E**	Linear mixed model (lmer)	HHD: Mean of all measurements of one leg<br>COMP: Mean of all measurements of COMP one leg on the same day (measuring day 2)	$y_{imj} = \beta_0 + \beta_{1,m(i)} + b_{j(i)}$<br>Y: HHD, m(i): COMP, j(i): animal ID corresponding to the ith observation
**F**	Linear mixed model (lmer)	HHD: Mean of all measurements of one leg<br>HFPS: Median of all measurements of HFPS of one leg on the same day (measuring day 2)	Y: HHD, m(i): HFPS, j(i): animal ID corresponding to the ith observation

HFPS: hind feet position score; DIG: digital protractor; COMP: compass app.

Model Nr.	Parameter	Estimate	Standard error	Df	t-value	Pr(>|t|)
**A**	Intercept	6.88	0.69	75.12	10.03	1.68e-15 ***
	Independent value	0.62	0.05	77.68	12.34	< 2e-16 ***
**B**	Intercept	9.02	0.64	93.53	14.18	< 2e-16 ***
	Independent value	4.16	0.41	98.72	10.09	< 2e-16 ***
**C**	Intercept	5.15	0.76	79.28	6.78	1.94e-09 ***
	Independent value	5.49	0.50	84.89	11.04	< 2e-16 ***
**D**	Intercept	0.74	0.14	102.00	5.45	3.57e-07 ***
	Independent value	0.00	0.00	102.00	- 0.21	0.836
**E**	Intercept	0.83	0.10	102.00	8.46	2.09e-13 ***
	Independent value	0.00	0.00	102.00	- 1.19	0.236
**F**	Intercept	0.73	0.08	102.00	8.64	8.09e-14 ***
	Independent value	-0.01	0.06	102.00	- 0.19	0.846

Significance codes: 0 – 0.001 = ***; 0.001 – 0.01 =**; 0.01 – 0.05 = *; 0.05 – 0.1 = not significant.
